# Zugang zur Elektrokonvulsionstherapie bei Menschen mit fehlender Einwilligungsfähigkeit und als Behandlung gegen den natürlichen Willen: Expertenkonsensus und Stellungnahme der Deutsche Gesellschaft für Psychiatrie und Psychotherapie, Psychosomatik und Nervenheilkunde (DGPPN)

**DOI:** 10.1007/s00115-025-01816-8

**Published:** 2025-03-05

**Authors:** David Zilles-Wegner, Jakov Gather, Alkomiet Hasan, Jürgen L. Müller, Thomas Pollmächer, Alfred Simon, Tilman Steinert, Alexander Sartorius

**Affiliations:** 1 Klinik für Psychiatrie und Psychotherapie, Universitätsmedizin Göttingen, Von-Siebold-Str. 5, 37075 Göttingen, Deutschland; 2 Klinik für Psychiatrie, Psychotherapie und Präventivmedizin, LWL-Universitätsklinikum, Ruhr-Universität Bochum, Bochum, Deutschland; 3 Institut für Medizinische Ethik und Geschichte der Medizin, Ruhr-Universität Bochum, Bochum, Deutschland; 4 Klinik für Psychiatrie, Psychotherapie und Psychosomatik, Medizinische Fakultät, Universität Augsburg, Augsburg, Deutschland; 5 Deutsches Zentrum für psychische Gesundheit (DZPG), München/Augsburg, Deutschland; 6 Schwerpunktprofessur für Forensische Psychiatrie, Universitätsmedizin Göttingen, Göttingen, Deutschland; 7 Asklepios Klinik für Forensische Psychiatrie Göttingen, Göttingen, Deutschland; 8 Klinikum Ingolstadt, Zentrum für psychische Gesundheit, Ingolstadt, Deutschland; 9 Klinik für Psychiatrie und Psychotherapie, LMU, München, Deutschland; 10 Akademie für Ethik in der Medizin, Universitätsmedizin Göttingen, Göttingen, Deutschland; 11 Klinik für Psychiatrie und Psychotherapie, ZfP Südwürttemberg, Universität Ulm (Weissenau), Ulm (Weissenau), Deutschland; 12 Klinik für Psychiatrie und Psychotherapie, Zentralinstitut für Seelische Gesundheit, Mannheim, Deutschland; 13 Deutsches Zentrum für psychische Gesundheit (DZPG), Mannheim, Deutschland

**Keywords:** Medizinethik, Einwilligungsfähigkeit, Forensische Psychiatrie, Zwangsmaßnahme, Zwangsbehandlung, Medical ethics, Decision-making capacity, Forensic psychiatry, Involuntary treatment, Nonvoluntary treatment

## Abstract

Die Elektrokonvulsionstherapie (EKT) ist ein klinisch bewährtes und evidenzbasiertes Verfahren zur Behandlung besonders schwerwiegender oder therapieresistenter psychiatrischer und neuropsychiatrischer Störungen. Aus den Indikationen zur EKT ergibt sich, dass ein relevanter Anteil der Patienten mit EKT-Indikation nicht einwilligungsfähig ist. Internationale und nationale Arbeiten zeigen, dass bei Betroffenen ohne Einwilligungsfähigkeit bzw. bei Behandlung gegen den natürlichen Willen (Zwangsbehandlung) restriktive Gesetze und Rechtsprechung die Anwendung der EKT erschweren oder verhindern können. Patienten mit Indikation zur EKT und fehlender Einwilligungsfähigkeit stellen eine vulnerable Personengruppe dar, für die in vielen Fällen keine gleichwertige Therapiealternative zur Verfügung steht. Die Entscheidung über eine EKT bei einwilligungsunfähigen Patienten, insbesondere gegen den natürlichen Willen, ist von hoher rechtlicher und medizinethischer Komplexität, weil je nach Einzelfall sowohl die Durchführung als auch das Unterlassen der Behandlung Grundrechte des Betroffenen verletzen kann. Die verfügbare Evidenz bei initial gegen den natürlichen Willen behandelten Patienten zeigt in der Summe gute Ansprechraten sowie identisch hohe retrospektive und zukünftige Zustimmungswerte zur Therapie im Vergleich zur Behandlung mit initialer Einwilligung.

Zusammen mit medizinethischen Erwägungen kommen die Autoren zu der Schlussfolgerung, dass für die EKT dieselben normativen Maßstäbe wie für alle anderen medizinischen Behandlungsverfahren gelten sollten. Dies gilt auch für eine mögliche Behandlung gegen den natürlichen Willen. Eine gesonderte und zum Teil restriktivere Handhabung im Vergleich zu anderen medizinischen Maßnahmen ist weder medizinisch noch ethisch gerechtfertigt. Strukturelle und juristische Hürden, die den Zugang schwer und manchmal lebensbedrohlich erkrankter Patientinnen und Patienten zu einer indizierten Behandlung erschweren, sollten hinterfragt und wo möglich und notwendig beseitigt werden.

## Abstract (en)

Electroconvulsive therapy (ECT) is a clinically well-established, evidence-based procedure for the treatment of particularly severe or treatment-resistant psychiatric and neuropsychiatric disorders. A considerable number of patients who require ECT are unable to provide informed consent due to their medical condition. Both international and national studies show that restrictive laws and legal rulings can hinder or even prevent the use of ECT in patients lacking the capacity to provide informed consent or in cases of nonvoluntary treatment (coercive treatment). Patients with indications for ECT who lack the capacity to consent constitute a vulnerable group, often with no viable alternative therapy available. The decision to administer ECT to individuals lacking the capacity to consent, particularly as a nonvoluntary treatment, is highly complex in terms of legal and medical ethics aspects because depending on the circumstances, both administering and withholding ECT can profoundly impact the patient’s fundamental rights. The available evidence shows that patients initially treated against their will exhibit good overall response rates, with equally high retrospective and prospective approval for therapy compared to patients who initially consented to treatment.

Together with the medical ethics considerations the authors conclude that the use of ECT should adhere to the same ethical and normative standards as all other medical interventions. This also applies to cases involving involuntary treatment. Adopting a more restrictive approach to ECT compared to other medical measures is neither medically nor ethically justified. Structural and legal barriers restricting access to necessary treatment for patients with severe and potentially life-threatening conditions should be critically reviewed and, when possible and necessary, removed.

## 1. Einleitung

Die Elektrokonvulsionstherapie (EKT) ist ein seit Jahrzehnten klinisch bewährtes und evidenzbasiertes Verfahren zur Behandlung besonders schwerwiegender oder therapieresistenter psychiatrischer und neuropsychiatrischer Störungen [1]. Aus den Indikationen zur EKT [2] ergibt sich nachvollziehbar, dass ein relevanter Anteil der Patienten mit EKT-Indikation nicht einwilligungsfähig ist. Internationale und nationale Arbeiten zeigen, dass bei Betroffenen ohne Einwilligungsfähigkeit [3] bzw. bei Behandlung gegen den natürlichen Willen (Zwangsbehandlung [4]) restriktive Gesetze und Rechtsprechung die Anwendung der EKT erschweren oder verhindern können. Patienten mit Indikation zur EKT und fehlender Einwilligungsfähigkeit stellen eine vulnerable Personengruppe dar, für die in vielen Fällen keine gleichwertige Therapiealternative zur Verfügung steht. Eine Entscheidung über eine EKT bei einem einwilligungsunfähigen Patienten, u. U. auch gegen seinen natürlichen Willen, ist von hoher rechtlicher und medizinethischer Komplexität, weil je nach den Gegebenheiten im Einzelfall sowohl die Durchführung als auch das Unterlassen einer EKT Grundrechte des Betroffenen erheblich verletzen kann.

## 2. Höchstrichterliche Entscheidungen

In den vergangenen Jahren gab es mehrere höchstrichterliche Entscheidungen zur Genehmigungsfähigkeit der EKT gegen den sog. natürlichen Willen einwilligungsunfähiger Personen (Zwangsbehandlung [5, 6]). Diese kamen in Abhängigkeit vom konkreten Fall zu unterschiedlichen Ergebnissen. Es seien Fälle denkbar, in denen die „Durchführung einer EKT gegen den Widerstand des Patienten kunstgerecht“ sein und sich als „Erfüllung der in Art. 2 Abs. 2 Satz 1 GG gründenden staatlichen Schutzpflicht“ darstellen könne, „hilfsbedürftigen Menschen (…) notfalls auch gegen ihren natürlichen Willen Schutz durch ärztliche Versorgung zu gewähren“ [6]. Auch eine von den Instanzgerichten genehmigte, ggf. langfristig notwendige Erhaltungs-EKT mit dem Ziel der Symptomlinderung wurde vom BGH bestätigt [7]. Der überwiegende Nutzen für die Betroffene wurde darin gesehen, „lebensbedrohliche Zustände“ zu verhindern sowie „Zustände, die mit ihrer Menschenwürde unvereinbar sind und ihren Anspruch auf Teilhabe am Leben in der Gemeinschaft faktisch entwerten.“

Prinzipiell legte der BGH in den Beschlüssen jedoch ein restriktives Regel-Ausnahme-Verhältnis zugrunde, nach dem die EKT bei entgegenstehendem natürlichem Willen in der Regel unterbleibe. Gestützt wurde dies auf die Formulierung in der Stellungnahme der Bundesärztekammer aus dem Jahr 2003, dass im Falle des Widerspruchs nicht einwilligungsfähiger Patienten im Regelfall auf die EKT verzichtet wird [8]. Neue, in der Zwischenzeit publizierte Primärliteratur (s. unten) blieb in den Beschlüssen unberücksichtigt.

2023 wurde ein Fall aus der forensischen Psychiatrie am Schweizer Bundesgericht verhandelt [9, 10]. Die Frage, ob eine EKT im vorliegenden Fall als medizinische Maßnahme gerechtfertigt sei, wurde vom Gericht nicht inhaltlich entschieden. Vielmehr stellte es fest, dass aus schon formalen Gründen keine EKT angeordnet werden könne, weil diese im (nach Schweizer Recht) maßgeblichen Strafurteil im Gegensatz zu einer medikamentösen Zwangsbehandlung nicht als mögliche Behandlungsform erwähnt wurde. In der Konsequenz wird dieser Patient voraussichtlich langfristig in einem hochgesicherten forensischen Setting inklusive Bewegungseinschränkung im Sinne von Fixierungsmaßnahmen verbleiben [10], obwohl mit der EKT eine evidenzbasierte und in einer S3-Leitlinie empfohlene Behandlung der ursächlichen Erkrankung verfügbar ist [11] und obwohl die behandelnde Einrichtung und ein Gutachter die EKT gleichermaßen als indiziert ansahen und schließlich der nicht einwilligungsfähige Patient zwar ambivalent war, die Behandlung aber nicht durchgehend ablehnte.

## 3. Zugang zur EKT und Konsequenzen der Nichtbehandlung

Ein erschwerter Zugang zur EKT für Patienten mit fehlender Einwilligungsfähigkeit ist international beschrieben und wird aus klinischer wie medizinethischer Sicht kritisiert [3], da dies zu einer Ungleichbehandlung solcher Patienten führt, Erfolg versprechende Behandlungen inklusive der Chance auf Wiederherstellung der Einwilligungsfähigkeit verhindert und durch die Nichtbehandlung zu weiteren gesundheitlichen Schäden führen kann. Eine Sonderstellung der EKT gegenüber anderen medizinischen Maßnahmen (z. B. somatische oder psychiatrische Medikation, Operationen) ist wissenschaftlich nicht begründbar, wenngleich den Autoren die teilweise andere, u. a. durch unsachgemäße und emotionale mediale Darstellungen geprägte öffentliche Wahrnehmung bewusst ist. In anderen Kontexten wurden die schwerwiegenden bis hin zu letalen Konsequenzen einer fehlenden Verfügbarkeit der EKT hinreichend beschrieben [12–16].

## 4. Evidenz zur EKT bei fehlender Einwilligungsfähigkeit und bei Behandlung gegen den natürlichen Willen

Nachfolgend wird die aktuell verfügbare wissenschaftliche Literatur zur EKT in der Behandlung nicht einwilligungsfähiger Patienten sowie als ärztliche Zwangsmaßnahme dargestellt. Für medizinische Maßnahmen gegen den natürlichen Willen kann es keine randomisierten kontrollierten Studien geben. Entsprechend müssen sich klinische Entscheidungen an den Ergebnissen anderer Studien orientieren. Die S3-Leitlinie Schizophrenie [11] empfiehlt die EKT bei eindeutiger medikamentöser Behandlungsresistenz mit dem Ziel der Verbesserung des klinischen Gesamtzustands. Der Empfehlungsgrad B reflektiert dabei die Sicherheit der Evidenz, nicht die Wirksamkeit der Behandlung [4]. Grundprinzip der evidenzbasierten Medizin ist, die verfügbare wissenschaftliche Evidenz mit klinischer Erfahrung und der Präferenz der betroffenen Personen in Beziehung zu setzen. Exemplarisch kann dies an der perniziösen Katatonie gezeigt werden, für welche die Leitlinie die EKT trotz fehlender hochwertiger Evidenz mit dem höchsten Empfehlungsgrad A empfiehlt [11]. Die Australische Praxisleitlinie differenziert ihre Empfehlung weiter aus und sieht einen Stellenwert für die EKT sowohl in der Behandlung der akuten Schizophrenie als auch bei Therapieresistenz [17]. Als evidenzbasierte Empfehlung wird die EKT (zusammen mit einem Antipsychotikum) empfohlen in Situationen, in denen ein rasches klinisches Ansprechen erforderlich ist, sowie bei therapieresistenter Schizophrenie und inadäquatem Ansprechen auf Clozapin.

Bei Menschen mit fehlender Einwilligungsfähigkeit fand eine Metaanalyse kontrollierter Beobachtungsstudien mit insgesamt 1299 Patienten ein gleichwertiges (Schizophrenie, 3 Studien, *n* = 386) bzw. sogar besseres (Depression, 6 Studien, *n* = 697) Ansprechen auf EKT im Vergleich zur diagnosespezifischen Kontrollgruppe mit Einwilligungsfähigkeit [18]. Hinweise auf unterschiedliche Nebenwirkungsraten zwischen den Gruppen gab es nicht. Auch Daten aus dem Schottischen Akkreditierungsnetzwerk SEAN zeigen, dass fehlende Einwilligungsfähigkeit bei Depression sogar mit einem besseren Ansprechen auf EKT assoziiert ist [19], was den prädiktiven Wert einer höheren Krankheitsschwere widerspiegelt [20]. Wichtig ist zu erwähnen, dass die Arbeiten [18, 19] eine nicht näher spezifizierte Anzahl an Patienten umfassten, die gegen den natürlichen Willen behandelt wurden, da dies in der internationalen Literatur nicht gegenüber einer Behandlung bei fehlender Einwilligungsfähigkeit abgegrenzt wird.

Die Literatur zur Anwendung der EKT gegen den natürlichen Willen ist daher im Vergleich deutlich begrenzter. Eine Fallserie beschrieb alle Patienten (*n* = 8), die über einen definierten Zeitraum an zwei Zentren gegen den natürlichen Willen mit EKT behandelt wurden [21]. 7 von 8 Patienten sprachen gut oder sehr gut auf die Behandlung an, inklusive einer Wiederherstellung der Einwilligungsfähigkeit und Zustimmung zur weiteren Behandlung im Verlauf. Eine Arbeit aus Schottland untersuchte die Patientensicht zustimmender und gegen den Willen behandelter Patienten [22]. Es zeigten sich keine Unterschiede hinsichtlich der Wirksamkeit und der zukünftigen Akzeptanz der Behandlung, nur 2 von 11 Patienten lehnten eine zukünftige Behandlung ab. Eine retrospektive Fragebogenstudie erfasste deutschlandweit Fälle von EKT gegen den natürlichen Willen [23]. Für 14 der 15 identifizierten Patienten wurde ein gutes klinisches Ansprechen beschrieben, bei 11 Patienten eine zumindest temporäre Wiederherstellung der Einwilligungsfähigkeit. 8 dieser 11 Patienten stimmten einer Fortführung der EKT zu. Zusammengefasst spricht dies unter den gegebenen methodischen Limitationen für hohe Ansprechraten, eine identisch gute Beurteilung der Therapie durch die Patienten im Vergleich zur Behandlung mit initialer Einwilligung, hohe Zustimmungsraten bezüglich zukünftiger Behandlungen und die realistische Chance auf Wiederherstellung der Einwilligungsfähigkeit.

## 5. Evidenz zur EKT im Maßregelvollzug

Die Frage nach der Rechtfertigung medizinischer Maßnahmen gegen den natürlichen Willen stellt sich auch und besonders im MRV. In forensischen Kliniken stellen Erkrankungen aus dem schizophrenen Formenkreis das häufigste Krankheitsbild dar [24]. Die untergebrachten Personen weisen in der Regel eine langjährige Vorgeschichte mit unbehandelten Symptomen und Adhärenzproblemen auf [25]. Therapieresistente Verläufe bei Schizophrenie sind mit einer längeren Unterbringungsdauer assoziiert, sodass der EKT als leitliniengerechter Maßnahme eigentlich ein besonderer Stellenwert in der Therapie zukommen sollte.

Im Gegensatz hierzu gibt es nur wenig empirische Daten zur Anwendung der EKT im MRV. Eine aktuelle systematische Übersichtsarbeit [26] identifizierte Fallberichte und eine Fallserie mit insgesamt 13 Patienten (8 Behandlungen mit Zustimmung, 4 Behandlungen gegen den natürlichen Willen, 1 unklar) und entsprechendem Risiko eines Publikationsbias. 11 der 13 berichteten Patienten sprachen trotz teilweise jahrelanger Krankheits- und Episodendauer gut auf EKT an. Neben der Symptomverbesserung wurde eine Reduktion des bei der Mehrzahl der Personen assoziierten aggressiven Verhaltens beschrieben, ebenso Verbesserungen des Funktionsniveaus sowie im Verlauf erfolgreiche Entlassungen aus dem forensischen Setting [27]. Eine Fragebogenstudie in Deutschland identifizierte für 2018 insgesamt 32 Menschen im MRV, die mit EKT behandelt wurden. Im klinischen Gesamteindruck wurde eine durchschnittlich gute Besserung beschrieben [28]. Detailliertere, noch unpublizierte Daten, die nach den aktualisierten Empfehlungen der letzten S3-Leitlinie Schizophrenie erhoben wurden, bestätigen die potenzielle Wirksamkeit der EKT im Setting des MRV. 16 von 29 Patienten sprachen im klinischen Gesamteindruck gut oder sehr gut auf EKT an, und es konnten im Kontext der EKT Lockerungen etabliert werden. Die Ergebnisse sprechen unter Berücksichtigung der methodischen Limitationen dafür, dass Wirksamkeitsdaten zur EKT bei Schizophrenie aus der Allgemeinpsychiatrie [11, 17, 29] auf den MRV übertragbar sein könnten.

## 6. Medizinethische Überlegungen

Bei Personen mit fehlender Einwilligungsfähigkeit ist es zunächst wichtig, Maßnahmen der Entscheidungsassistenz zu ergreifen und zu versuchen, die Einwilligungsfähigkeit der betroffenen Person zu fördern, sodass sie zu einer selbstbestimmten Entscheidung für oder gegen eine EKT-Behandlung in der Lage ist [30]. Gelingt es trotz Entscheidungsassistenz nicht, die Entscheidungsfähigkeit wieder herzustellen, muss geprüft werden, ob die Person zu einem früheren Zeitpunkt im Zustand der Einwilligungsfähigkeit in einer Patientenverfügung oder Behandlungsvereinbarung Festlegungen zu einer zukünftigen Behandlung getroffen hat [31] und ob diese auf die aktuelle Lebens- und Behandlungssituation zutreffen. Wenn solche zutreffenden Festlegungen getroffen wurden, dann sind sie bindend, egal, ob sich der Betreffende für oder gegen eine Behandlung ausgesprochen hat. Die DGPPN hat kürzlich ein Formular [32] für eine Patientenverfügung für den Bereich der psychischen Gesundheit entwickelt, in dem u. a. Angaben zu einer zukünftigen EKT-Behandlung gemacht werden können. Liegt keine Vorausverfügung vor, muss der mutmaßliche Wille der Person ermittelt werden, also welche Entscheidung die betroffene Person in einer gegebenen Situation – in diesem Fall im Hinblick auf eine medizinisch indizierte EKT – vor dem Hintergrund früherer Aussagen sowie ihrer persönlichen Wertvorstellungen und Präferenzen am ehesten getroffen hätte, wenn sie einwilligungsfähig gewesen wäre [33].

Widerspricht eine einwilligungsunfähige Person einer EKT, stellt sich die Frage, ob eine Behandlung auch gegen den natürlichen Willen (Zwangsbehandlung) indiziert und ethisch gerechtfertigt ist. Bei der Beurteilung dieser Frage sind aus ethischer Sicht an die Zwangsbehandlung mittels EKT dieselben Kriterien anzulegen wie an jede andere psychiatrische oder somatische Zwangsbehandlung – und zwar unabhängig vom jeweiligen klinischen Setting (Allgemeinpsychiatrie oder Maßregelvollzug). Für eine ethische Rechtfertigung einer Zwangsbehandlung ist es weder hinreichend, dass eine medizinische Indikation für die Maßnahme besteht, noch dass die Maßnahme dem vorausverfügten oder mutmaßlichen Willen der Person entspricht. Ergänzend ist in jedem einzelnen Fall zu prüfen, ob es sich bei der Zwangsbehandlung um eine geeignete, notwendige und verhältnismäßige Maßnahme handelt [30, 33]. Dabei muss vor allem geklärt werden, ob es mildere Alternativen zur Zwangsbehandlung mittels EKT gibt und ob der Nutzen das Risiko der Maßnahme deutlich überwiegt. In diese Abwägung geht zunächst die Evidenz für die beabsichtigte Behandlung ein. Im Fall der EKT ist sie, wie oben dargestellt, auch bei Psychosen generell gut, allerdings zu ergänzen um die besondere Betrachtung des Einzelfalls (z. B. Wirkung früherer Behandlungen). Auch müssen Art, Ausmaß und Dauer des voraussichtlich erforderlichen Zwangs als potenzieller Schaden für die psychische Gesundheit in die Abwägungen eingehen [34]. Eine voraussichtlich relativ kurz dauernde Maßnahme bei nur allgemein negativistisch gezeigter Ablehnung wird man bei der Abwägung der Umstände des Einzelfalls eher befürworten als eine Durchführung unter Anwendung massiven körperlichen Zwangs bei einer Person, die zu erkennen gibt, dass sie sich dadurch in ihren Persönlichkeitsrechten zutiefst verletzt fühlt. Andererseits müssen auch die Risiken berücksichtigt werden, wenn einer einwilligungsunfähigen Person eine potenziell sehr wirksame und verfügbare Behandlung vorenthalten wird, z. B. im Hinblick auf eine weitere Chronifizierung oder Verschlechterung der Erkrankung, subjektive Symptombelastung (z. B. durch Angst und psychomotorische Unruhe), ggf. notwendige andere (z. B. pharmakologische) Zwangsmaßnahmen oder einen langfristigen Freiheitsentzug aufgrund andauernder krankheitsbedingter Eigen- oder Fremdgefährdung.

Vor dem Hintergrund der Komplexität der ethischen Abwägung sollte die Entscheidungsfindung im multiprofessionellen Team unter Einbeziehung des rechtlichen Vertreters, z. B. im Rahmen einer ethischen Fallbesprechung [35–37], erfolgen. Auch im Falle einer genehmigten Zwangsbehandlung soll versucht werden, eine bestmögliche Akzeptanz der Betroffenen zu erreichen und die Durchführung für alle Beteiligten vertretbar auszugestalten.

## 7. Schlussfolgerung

Strukturelle und juristische Hürden, die den Zugang schwer und manchmal lebensbedrohlich erkrankter Patientinnen und Patienten zu einer indizierten Behandlung erschweren, sollten hinterfragt und wo möglich und notwendig beseitigt werden.

Die begrenzte, im Ergebnis jedoch konsistente Evidenz sowie die klinische Erfahrung der ärztlich tätigen Autoren spricht dafür, dass die EKT auch als Maßnahme gegen den natürlichen Willen eine wirksame und verträgliche Behandlung darstellt, deren Anwendung nicht zuletzt durch die realistische Chance einer Wiederherstellung der Einwilligungsfähigkeit sowie die zumeist gute retrospektive Bewertung durch die Betroffenen gerechtfertigt werden kann.

Dies führt zusammen mit medizinethischen Erwägungen zu der Schlussfolgerung, dass für die EKT dieselben normativen Maßstäbe wie für alle anderen medizinischen Behandlungsverfahren gelten sollten. Dies gilt auch für eine mögliche Behandlung gegen den natürlichen Willen. Eine gesonderte und zum Teil restriktivere Handhabung im Vergleich zu anderen medizinischen Maßnahmen ist weder medizinisch noch ethisch gerechtfertigt.
